# Aquaporin-4 protein expression in normal canine brains

**DOI:** 10.1186/s12917-021-02745-9

**Published:** 2021-01-18

**Authors:** Patricia Álvarez, Ester Blasco, Martí Pumarola, Annette Wessmann

**Affiliations:** 1 Neurology and Neurosurgery Service, Pride Veterinary Centre, Derby, UK; 2 Unit of Murine and Comparative Pathology (UPMiC), Department of Animal Medicine and Surgery, Veterinary Faculty, Autonomous University of Barcelona, Barcelona, Spain; 3 Center on Bioengineering, Biomaterials and Nanomedicine (CIBER-BBN) Networking Research, Universitat Autònoma de Barcelona, Bellaterra, Barcelona, Spain

**Keywords:** Aquaporin-4, Brain, Dog, Grey matter, Immunohistochemistry, Non‐pathological, White matter, Brain development

## Abstract

**Background:**

Aquaporin-4 (AQP4) is in growing recognition as potential marker for cancer progression, differentiation and therapeutic intervention. No information is available about AQP4 expression in the normal canine brain. The aim of this histopathological study is to confirm the presence of AQP4 by immunohistochemistry technique in a group of non-pathological canine brains and to describe its expression and distribution across the brain.

**Results:**

Twelve non-pathological canine brains of various ages (ranging from 21 days to 17 years) and breeds were included in the study. Immunohistochemical expression of AQP4 was analyzed using formalin-fixed paraffin-embedded brain tissue sections. The findings were correlated between AQP4 expressing cells and astrocytes using glial fibrillary acidic protein (GFAP). AQP4 expression was more marked in the astrocyte foot processes of subpial, perivascular and periventricular surfaces in all specimens. The majority of the canine brain sections (9/12) presented with an AQP4 predilection for white matter tracts. Interestingly, the two youngest dogs (21 days and 3 months old) were characterized by diffuse AQP4 labelling in both grey and white matter tracts. This result may suggest that brain development and ageing may play a role in the AQP4 distribution throughout the canine brain.

**Conclusions:**

This is the first study to describe immunohistochemical distribution of AQP4 in normal canine brains. The AQP4 expression and distribution in non-pathological canine brains was comparable to other species. Larger studies are needed to substantiate the influence of breed and ageing on AQP4 expression in the normal canine brain.

## Background

Aquaporins (AQPs) are a family of transmembrane water-channel proteins distributed in membranes of various biological cells of animals and humans mainly facilitating transport of water between cells [1]. One of the most prominent aquaporins in the central nervous system (CNS) is Aquaporin-4 protein (AQP4) [1]. AQP4 plays a crucial role in maintaining water homeostasis, cell migration and neuroexcitation in the brain [1]. The AQP4 expression in the CNS has been associated with neuroinflammatory conditions such as neuromyelitis optica spectrum disorders, tumor types and grades, tumor proliferation, migration, angiogenesis and tumor-associated edema in people [2–6].

Most information about CNS AQP4 is available in humans, rats and mice [7, 8]. In normal circumstances, AQP4 is expressed more abundantly in the astrocyte foot processes of the perivascular, subpial and subependymal areas and in the basolateral membrane of ependymal cells. These locations are special sites of major fluid transport where AQP4 is responsible for water balance regulation in and out the CNS [1]. Similar locations have been demonstrated in other species including *Macaca Fascicularis* and *Psittacus erithacus* [9, 10].

Only scarce information is available about AQP4 distribution in diseased canine brains. Klemens et al. [11] described the expression of AQP4 in canine cerebellar samples of canine distemper virus infected dogs. This study showed that there was a more severe loss of AQP4 in acute distemper lesions when compared to subacute or chronic lesions in these dogs. Spitzbarth et al. [12] analyzed AQP4 expression using tissue microarray technique searching for AQP4 expression patterns in different types of canine CNS neoplasms and highlighted AQP4 as a novel marker helping to discriminate between canine astrocytoma and oligodendroglioma. Considering the growing recognition of AQP4 in the CNS and its potential diagnostic and therapeutic implications, knowledge about the distribution of AQP4 in normal canine brain is urgently needed as basis for future studies. The purpose of this study is first, to confirm the presence of AQP4 in the normal canine brain using immunohistochemistry technique and secondly, to describe the AQP4 distribution in non-pathological brains in a group of dogs of different age and breed (Table 1).

**Table 1:** Canine brains included in this study

Case Number	Breed	Age
N01	Cross breed	21 days
N02	Bichon Maltese	3 months
N03	White Swiss Shepherd dog	3 months
N04	Cocker Spaniel	5 months
N05	Pitbull	3 years
N06	Cocker Spaniel	4 years
N07	Miniature Schnauzer	5 years
N08	Bull Terrier	5 years
N09	Cross breed	13 years
N10	Yorkshire Terrier	14 years
N11	Scottish Terrier	15 years
N12	Cross breed	17 years

## Results

AQP4-expressing cells were confirmed using immunohistochemistry in all canine brains included in this study. The expression of AQP4 in our canine population was examined by semiquantitative descriptive analysis. The complete data is contained in Table 2. AQP4 immunohistochemistry was co-localized with GFAP immunohistochemistry confirming the common astrocytic origin.

**Table 2:** Expression of AQP4 immunohistochemistry in the 12 canine brains following the semiquantitative classification system

		N01	N02	N03	N04	N05	N06	N07	N08	N09	N10	N11	N12
Neocortex^a^	GM	++	++	+/++	-/+	-/+	n/a^i^	-/+	-/+	-/+	-/+	-/+	+/++
	WM	+++	+++	+++	+++	+++	n/a	++	+++	+++	+++	+++	+++
Basal nuclei^b^	GM	++	++	-/+	-/+	-/+	n/a	+/++	+/++	-/+	+	+	++
	WM	++	++	++	++	+++	n/a	++	++	++	++	++	++
Archicortex^c^	GM	++	++	+	n/a	+/++	-/+	-/+	+/++	+	n/a	+	++
	WM	+++	+++	++	n/a	++	-	-	-/+	++	n/a	+++	+++
Paleocortex^d^		++	++	n/a	+	n/a	n/a	++	++	+/++	+++	++	+/++
Diencephalon^e^	GM	++	++	-/+	n/a	-/+	n/a	-	-	n/a	-	+	+
	WM	+++	+++	++	n/a	++	n/a	++	++	n/a	+	+++	+++
Midbrain Pons^f^	GM	++	++	n/a	+/++	+	+	-/+	-	-/+	-	-	-
	WM	+++	+++	n/a	+++	++	++	++	++	++	+/++	++	+/++
Medulla oblongata^g^	GM	++	++	n/a	+/++	+	-/+	-	-/+	-/+	-	-/+	-
	WM	+++	+++	n/a	+++	+++	++	++	++	+++	+++	++	++
Cerebellum^h^	GM	++	++	n/a	++	+/++	-/+	-	-/+	-/+	-	-	+/++
	WM	+++	+++	n/a	+++	+++	++	++	+++	+++	+++	+++	+++

Abbreviations: *GM *Grey matter, *WM *White matter, *n/a *Not applicable; -, negative; +, mild staining; ++, moderate staining; +++, strong staining; Neocortex^a^: frontal, parietal and temporal areas and subcortical white matter and corpus callosum; Basal nuclei^b^: Striatum body and internal capsule; Archicortex^c^: hippocampal formation, fimbria and fornix; Paleocortex^d^: piriform lobe; Diencephalon^e^: epithalamus (habenula nuclei), thalamic nuclei and white matter tracts; Midbrain/pons^f^: oculomotor nuclei, red nuclei, substantia nigra, periaqueductal grey matter and white matter tracts (crus cerebri, cerebellar tracts, transverse pontine fibers); Medulla oblongata^g^: caudal cranial nerves nuclei, white matter tracts (pyramids, medial longitudinal fasciculus, trapezoid body); Cerebellum^h^: cerebellar cortex and subcortical white matter; n/a^i^: Not applicable, corresponds to an incomplete evaluation due to the lack of tissue or low immunoreaction

**Fig. 1:**
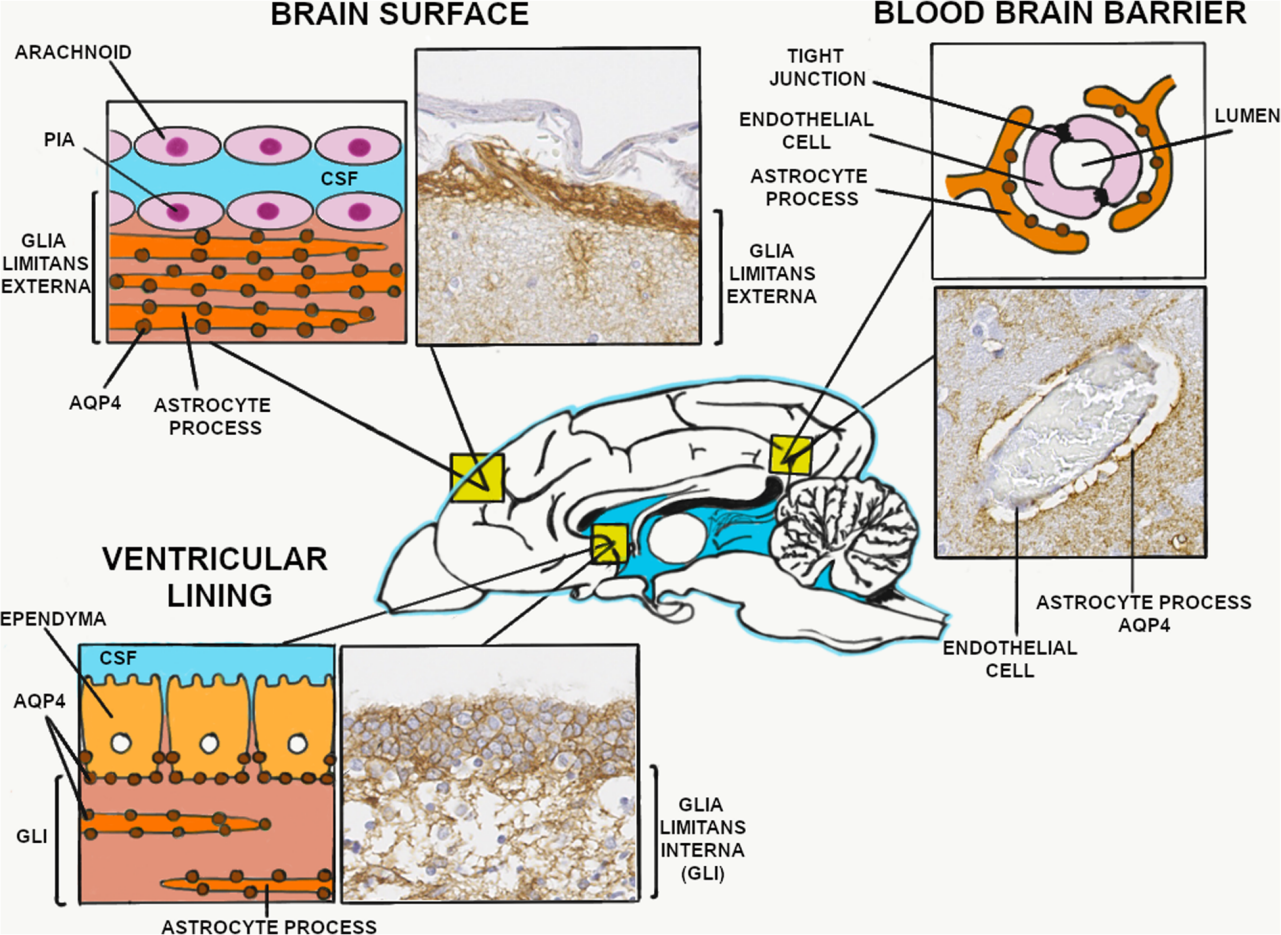
The immunohistochemistry of the main areas of AQP4 expression are represented with a schematic illustration (modified from Papadopoulos et al [1]): Brain surface-glia limitans externa, Ventricular lining-glia limitans interna and blood brain barrier

All twelve canine brains showed the greatest AQP4 immunoreaction in the plasma membrane of the perivascular astrocyte foot processes in a branching-fibrillar pattern. The glia limitans externa, located between the CNS parenchyma and the pia mater, showed a well-defined homogenous monolayer labeling. The periventricular surfaces also displayed a strong positive immunoreaction in the basolateral membrane of the ependymal cells and the associated bands of the subependymal astrocyte foot processes in a characteristic branching pattern. The intensity of the labeling was gradually fading towards the neuropil. APQ4 immunoreaction was absent in choroid plexus cells, neurons or other glial cells aside from astrocytes. The common areas of greater AQP4 expression in the canine brain are illustrated in Fig. 1.

Nine out of 12 dogs (N03-N11) showed a clear distinction between the grey and white matter. A predilection for AQP4 immunoreaction was concentrated in the white matter tracts and the subcortical cerebral transition area between the grey and white matter. A longitudinal pattern alongside nerve fibers involving mostly the white matter was identified in the subcortical white matter, corona radiata, internal capsule and corpus callosum. The basal nuclei showed variable expression from absent to mild patchy and punctuated immunoreaction surrounding the vascularization. Interestingly, the 2 youngest dogs (21 days old N01, 3 months old N02, 2/12) showed a slightly different presentation. All encephalic structures examined for N01 and N02 (2/12) (Table 2) were characterized by a generalized homogenous strong immunoreaction involving both grey and white matter. In the neocortex of these 2 dogs, AQP4 expressing cells were subjectively more concentrated in the deeper laminae of the cortical grey matter and located primarily surrounding blood vessels of any size in a branching-fibrillar pattern. To a lesser extent, immunoreaction was also present in the perineuronal astrocyte processes. The canine brain N12 (17 years old) showed a well-distinguished variation compared to the majority of the population (N03-N11). A greater and diffuse AQP4 immunoreaction was identified in the grey matter of the neocortex of the frontal and temporal areas.

The archicortex was largely positive for AQP4 expression in all canine brains (N01-N12). The labeling was denser in the inner margin of the dentate gyrus and external margin towards the meninges in the Cornu Ammonis surrounding the neuronal bodies. Interestingly, only in the canine brain N12 (17 years old) the degree of AQP4 immunostaining around the Cornu Ammonis was increased.

The paleocortex (piriform lobe) and parahippocampal gyrus presented a moderate diffuse and homogeneous labelling in all cases (N01-N12).

The brainstem showed a similar pattern in 9 canine brains (N03-N12) with a clear predilection for white matter tracts. The mesencephalon, the substantia nigra and interpeduncular nuclei had partial immunoreaction. The red nuclei and oculomotor nuclei were clearly less positive. At the pons and medulla oblongata, immunoreaction appeared to be denser in the white matter tracts at the level of the cerebellar peduncles, crus cerebri, transverse pontine fibers and pyramids.

The cerebellar cortex showed a similar immunoreaction in 9 specimens (N03-N11). AQP4 expressing cells exhibited a predilection for the cerebellar white matter. A low AQP4 immunoreaction was sporadically accumulated in the Purkinje layer in a perineuronal pattern and in the glomeruli of the granular layer. In the canine brain N12, a linear labeling of the molecular layer of the cerebellar cortical grey matter was present contrary to the common findings.

The habenular nuclei was only examined in three specimens of varied age including a young (N02, 3 months old), adult (N08, 3 years old) and senior dog (N11, 15 years old) and showed an equally strong AQP4 expression and diffuse distribution.

**Fig. 2:**
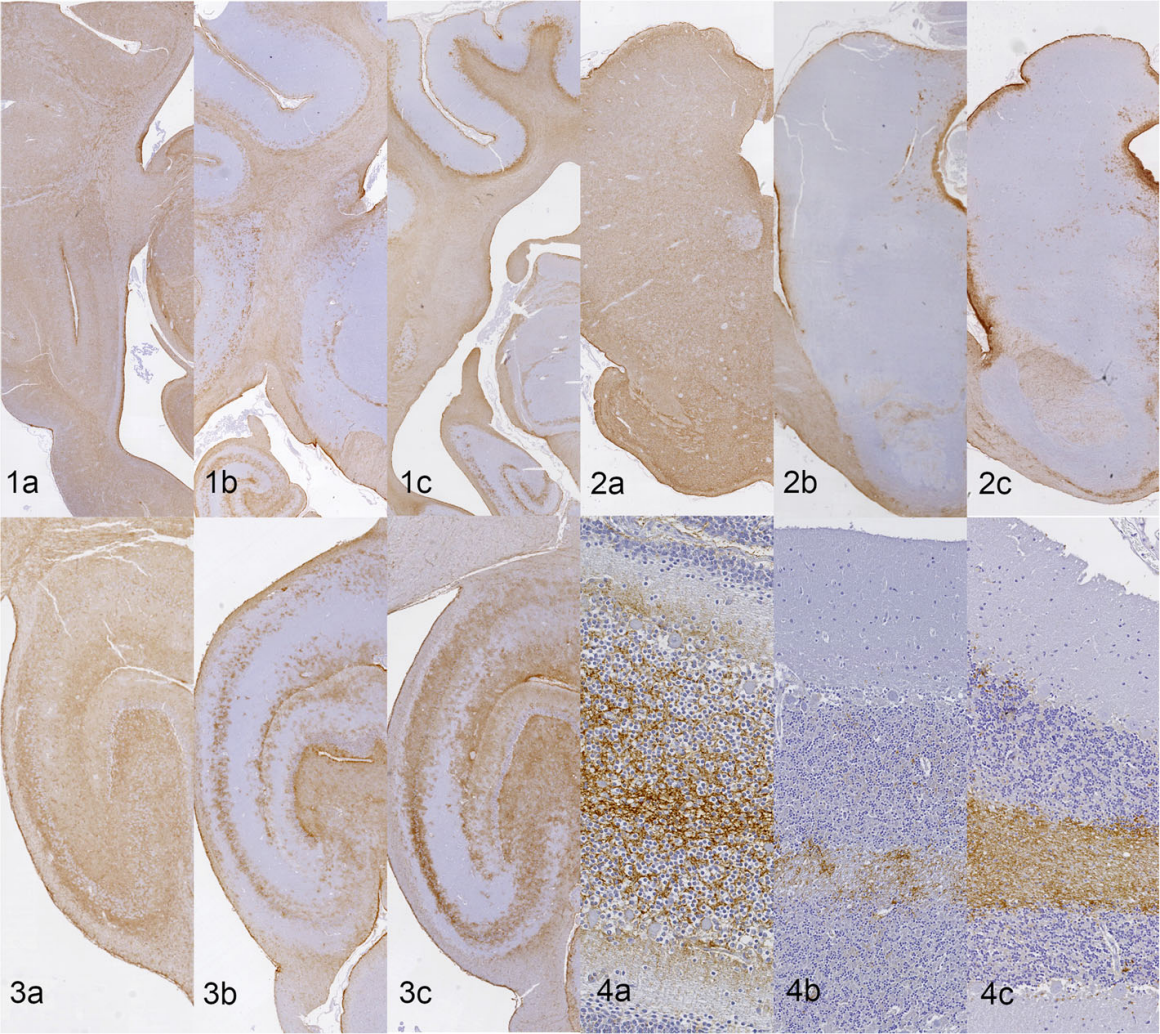
CNS AQP4 immunoreactivity comparison between dogs of varied ages at a different representative encephalic structure in transverse sections. Parietal/temporal cortex (1) (0.5 times magnification), Midbrain/pons (2) (0.6-1x), Archicortex with hippocampal formation (3) (2-2.5 times magnification) and Cerebellar cortex (4) (10-15 times magnification). The youngest dogs (a: N01 and N02) showed a generalized AQP4 labeling diffusively involving the neuroparenchyma. The adult and senior dogs (b and c: N05,N06, N10 and N11) presented greater expression of AQP4 in the subcortical WM and at the interface between major fluid compartments (periventricular and subpial surfaces). The hippocampal distribution in b and c was concentrated in the GM of the dentate gyrus and Amon’s horn

 A comparison of the AQP4 immunohistochemistry distributions in the above described representative encephalic structures of dogs of varied ages is illustrated in Fig. 2.

## Discussion

This is the first study to confirm the expression and distribution of AQP4 using immunohistochemistry in normal canine brains. Previous veterinary publications have exclusively investigated the presence of CNS AQP4 immunohistochemistry in pathological conditions [11, 12], but no information was available so far in non-pathological canine brains. This finding supports the assumption that AQP4 plays also in the normal canine brains a crucial role in maintaining water homeostasis, cell migration and neuroexcitation [1].

The distribution of AQP4 showed many similarities to reports of other species. All examined canine brains were characterized by the strongest immunoreaction in the plasma membrane of astrocytes, particularly in the astrocytic foot processes of the perivascular space, the subpial and subependymal areas, and in the basolateral membrane of the ependymal cells, which are specific regions of major fluid transport. This is a characteristic distribution comparable to other species and supports the assumption that AQP4 plays also in the canine a major role as a water-channel protein maintaining the perivascular volume and cerebral blood perfusion as previously reported in other species [7, 9, 10, 13, 14].

A specific polarized distribution of AQP4 in astrocytes towards the astrocytic foot processes was seen in our canine population. A specific anchoring mechanism is found in this location which is called the dystrophin-associated protein complex [15]. The dystroglycan was found to interact with laminin and dynamin, which is necessary for the regulation of AQP4 internalization and therefore explaining the asymmetric enrichment towards perivascular astrocyte foot processes [16].

AQP4 immunoreaction was further absent in neurons, other glial cells and choroid plexus in our canine population. This was observed similarly in other species too [7–9, 14], apart from a reported low expression of AQP4 in the choroid plexus in rat and human brain tissue [4, 13].

A moderate diffuse homogeneous AQP4 immunostaining was seen in the paleocortex throughout our canine population, whereas a more concentrated labelling in the neocortex towards the subcortical white matter and the transition area between the grey and white matter was seen in the majority of the specimens (9/12). It can be suspected that this AQP4 distribution is related to the primitive origin of the paleocortex compared to the neocortex. The paleocortex has 3 cortical grey matter layers with its white mater located in an external disposition, whereas the neocortex comprises 6 layers of cortical grey matter with the white matter located in a subcortical disposition [17].

The hippocampal formation presented with increased AQP4 immunostaining in the dentate gyrus and Cornu Ammonis in all the canine brains of this study. This is not unexpected considering published findings in the mice brain where AQP4, was shown to be associated with the facilitation of rapid water fluxes required for maintaining K + homeostasis during electrical activity in same regions [18]. Thus, the results of our study suggests a similar significance in canine brains.

The distribution of AQP4 towards the white matter was evident in the majority of dogs (N03-N11, aged between 3 months and 15 years). Specifically, the expression was located in the subcortical white matter of both neocortex and cerebellum. The immunoreaction pattern was parallel to the nerve fibers. It is suspected that this particular distribution might play a role in maintaining intercellular junctions at the nodes of Ranvier [7]. Further, the white matter is characterized by a considerable concentration of fibrillary astrocytes compared to the grey matter [8]. Fibrillar astrocytes have highly branched processes compared to protoplasmic astrocytes [19]. It is considered that this distinct characteristic may account for the higher concentration of positive labeling within the white matter. On the contrary, monkeys and adult rats have been described to have a greater AQP4 expression in the grey matter instead [9, 13]. One of their explanations was its higher concentration of blood vessels compared to the white matter [9]. Although we did not find increased AQP4 expression in the grey matter compared to the white matter in our study, increased AQP4 labelling was additionally concentrated in the grey/white matter transition in most of the canine brains (9/12). The latter could be explained by a higher concentration of capillaries and perivascular astrocyte foot processes expressing AQP4 in this transition zone [20]. Moreover, the grey and white matter transition area is where a clearer distinction between protoplasmic versus fibrillary astrocytes is present, which may account for the presence of AQP4 immunostaining yet less strong compared to the white matter alone.

There was an AQP4 distribution tendency towards the white matter with increasing age in our study population. The 2 youngest canine brains (21 days old N01, 3 months old N02) showed a generalized and homogenous AQP4 immunostaining in both, the grey and white matter, across the brain. The reasoning behind this particular finding is uncertain. Previous studies in rats have explored whether increased AQP4 expression in this area is needed in the formation of the blood brain barrier during the brain development and the control of the perivascular volume [21, 22]. As seen in the developing mouse brain, AQP4 immunoreactivity varies between brain regions according to neuronal differentiation [21]. This suggests that the homogeneous distribution of AQP4 along the brain grey and white matter structures in two of the youngest canine dogs (N01, N02) may be related with postnatal brain development, neuronal migration, blood brain barrier formation and the need of an adequate hydrated milieu to favor the neuronal shifts. Whereas the distribution of AQP4 towards the white matter seen in the majority of the canine population, could be associated with AQP4’s primary role as water channel protein and therefore might be more limited to regions of actual fluid exchange, such as perivascular (blood), subpial and periventricular (CSF) areas.

A different distribution pattern was seen in the canine brain N12 when compared to the majority of the studied canine brains (9/12). The AQP4 immunoreaction was increased in the grey matter (cerebral frontal, parietal and cerebellar cortices and Cornu Ammonis) compared to the white matter. One possible explanation may be contemplated based on findings about dementia in people [23]. Owasil et al. [23] concluded that the expression of AQP4 was associated with astrocytes in the brain, and that the distribution of these astrocytes in the grey and white matter was correlated with the patient’s age and the severity of cerebral amyloid angiopathy. Here, demented patients seemed to have a higher concentration of AQP4 labeling in the cortical grey matter compared to non-demented patients [23]. Interestingly, the case N12 was the oldest dog with 17 years of age. In dogs, the diagnosis of canine cognitive dysfunction syndrome is based on behavioral signs and exclusion of other medical conditions [24]. Further, it is known clinically that the prevalence and severity of canine cognitive dysfunction increases with the age [25]. Different publications have studied that dogs affected by progressive cognitive impairment share certain histopathological changes including alterations related to the structure of certain proteins as in Alzheimer disease: neuronal loss, astrocytosis, amyloid-β deposition and rarely neurofibrillary tangles [26, 27]. As AQP4 is more abundantly expressed in astrocytes, AQP4 concentration is likely increased in diseases resulting in astrocytosis, which is a hallmark of neuroinflammation. Thus, the AQP4 overexpression seen in this dog (N12) raises the suspicion that preclinical cognitive dysfunction might have been present. Further studies would be needed to investigate the influence of canine cognitive dysfunction syndrome on the AQP4 distribution in the brain compared to senile brains and further, to possibly investigate AQP4 as a potential immunomarker for early detection of canine cognitive dysfunction syndrome. We cannot exclude the possibility that the increased grey matter AQP4 labelling noted in this particular brain (N12) could have also been an individual variant. Publications report a greater AQP4 expression in the grey matter of rats and monkeys, yet this is not described for the dog [9, 13].

The habenula was examined in 3 cases in which AQP4 immunostaining was found to be homogeneous despite the varied age (3 months to 15 years). The habenula is located in the dorsomedial border of the thalamus close to the pineal gland, which is forming part of a group of specialized structures defined as circumventricular organs [28, 29]. The circumventricular organs are located mainly at the midsagittal line around the third and fourth ventricles, often protruding into the lumen [30]. The singular characteristic of the circumventricular organs is the lack of blood brain barrier due to the presence of fenestrated capillaries [30]. They are recognized to play important integrative roles in the regulation of fluid and mineral balance [31]. This is not completely new. AQP4 expression was already demonstrated in astrocytes of other canine circumventricular organs such as the area postrema, and thus supporting the important contribution of this water-channel in the maintenance of the homeostasis [30].

Limitations of this study are the limited number of canine brains of various ages and breeds. The aim of the current study was to confirm the presence of AQP4 in non-pathological brains and to identify tendencies of AQP4 distribution in the canine brain, which was achieved. However, the limited number of cases can only give an idea about the wider dog population. Larger scale studies would be ideal to focus on particular aspects of AQP4 expression in normal canine brains such as particular ages (young or senile group) or possible breed specific findings. Another limitation of this study is the lack of clinical data. Inclusion criteria were brains defined as ‘non-pathological’ based on histopathological examination only. More clinical information would have been ideal to investigate our hypothesis that the unusual AQP4 distribution in the oldest dog (N12, 17 years old) was caused by early or preclinical canine cognitive dysfunction syndrome.

## Conclusions

This is the first study to confirm the presence AQP4 in non-pathological canine brains and to describe its distribution in normal canine brains using immunohistochemistry. APQ4 expression and distribution in the canine brain was comparable to other species including humans. AQP4 was widely expressed in the astrocyte cell plasma membrane, particularly in the foot processes of the blood brain barrier and at the border between the CNS and the cerebrospinal fluid. A particular redistribution tendency was observed for AQP4 expression with ageing from grey matter to white mater in most dogs. Brain development as well as ageing may affect AQP4 distribution throughout the canine brain.

Knowledge about AQP4 expression and distribution in normal canine brains will aid the understanding of the importance of abnormal AQP4 expression in canine brains with different pathological conditions.

## Methods

### Animals and specimen collection

A total of 12 canine brains were retrospectively evaluated. The dogs died or were euthanized due to causes unrelated to the CNS and donated to the Unit of Murine and Comparative Pathology, Autonomous University of Barcelona, Spain. Owner agreement was obtained at the time of the donation of the canine body. Post-mortem examination and harvesting of the brains for histopathological analysis were performed within 24 hours after death. Throughout this time, the animal bodies were refrigerated. A complete post-mortem examination including gross and histopathological analysis of the bodies was performed. Thorough examination of formalin-fixed paraffin-embedded transverse sections of the brains, using a rotatory microtome, followed by hematoxylin-eosin staining confirmed the lack of significant abnormalities. The age ranged between 21 days to 17 years (mean 6.4 years). Different canine breeds were included. Detailed information about the dogs is listed in Table 1.

Representative sections of the brain were evaluated at the level of the frontal cortex/corpus striatum, parieto-temporal cortex/diencephalon, mesencephalon, cerebellum/pons and medulla oblongata.

### Histology procedure

Samples of all tissues were fixed in 10% buffered formalin and paraffin-embedded.

Sections 3-µm thick of paraffin-embedded brain tissue of different selected areas were stained using standard hematoxylin-eosin dye. Primary antibodies specific for AQP4 and GFAP were used to characterize the distribution of the AQP4 and the astrocytes within the brain. Sections 3‐µm thick were mounted on capillary glass slides, dewaxed, and rinsed with water and treated with 3% peroxide hydrogen for 35 minutes to inhibit endogenous peroxidase activity. When antigen retrieval was necessary (GFAP), sections were heated for 20 minutes in a bain-marie at 98ºC with 10 mM citrate buffer pH 6.0, cooled for 30 minutes at room temperature (RT), and rinsed in phosphate‐buffered saline (PBS). Nonspecific binding was blocked with 30% normal goat serum diluted in PBS for 1 hour at RT. The samples were incubated overnight at 4 °C with primary antibodies: rabbit anti-AQP4 antibody (1:800, Chemicon, Temecula, CA), and rabbit anti-GFAP antibody (1:1000, Dako, Denmark). The sections were then rinsed with PBS and incubated for 40 minutes at RT with a labeled polymer according to the manufacturer’s instructions (Labeled Polymer—Dako REAL Envision-HRP K011, Dako, Denmark). Staining was completed by 10-minute incubation with 3, 3′-diaminobenzidine and counterstaining in hematoxylin for 3 seconds. For the negative control, an isotype-specific immunoglobulin was used as a substitute for the primary antibody in all experiments; no immunostaining was detected in these sections.

### Analysis

Microscopic description and semiquantitative evaluation of the expression and intensity of AQP4 immunoreaction was performed. The observer (PA) was not blinded to the signalment and clinical history of the canine study population. The AQP expression was distributed according to the following scale: (-) absence of detectable staining, (+) mild staining, (++) moderate staining in focal or diffuse patterns and (+++) strong staining (Table 2). This scale was achieved by assigning the strongest staining intensity (+++) to the subpial and subependymal areas. The staining intensity became progressively fainter (++,+, -) the further away from the periventricular and perivascular regions. Thereafter, the above-named other representative encephalic structures were compared to the staining intensity seen in the subpial and subependymal areas and the staining intensity was attributed accordingly. This pattern was found to be repeatable in all examined canine brains. The analysis was performed within the same day and was repeated up to three times to ensure consistency of the results.

## Data Availability

The datasets used and/or analyzed during the current study are available from the corresponding author on reasonable request.

## Abbreviations

AQP4: Aquaporin-4
AQPs: Aquaporins
CNS: Central nervous system
GFAP: Glial fibrillary acidic protein
GM: Grey matter
PBS: Phosphate-buffered saline
RT: Room temperature
WM: White matter
